# Long-term follow-up after rib fixation for flail chest and multiple rib fractures

**DOI:** 10.1007/s00068-018-1009-5

**Published:** 2018-09-18

**Authors:** Reinier B. Beks, Mirjam B. de Jong, Roderick M. Houwert, Arthur A. R. Sweet, Ivar G. J. M. De Bruin, Geertje A. M. Govaert, Karlijn J. P. Wessem, Rogier K. J. Simmermacher, Falco Hietbrink, Rolf H. H. Groenwold, Luke P. H. Leenen

**Affiliations:** 1 Department of Surgery, University Medical Center Utrecht, PO Box 85500, 3508 GA Utrecht, The Netherlands; 2 Utrecht Traumacenter, Utrecht, The Netherlands; 3 Julius Center for Health Sciences and Primary Care, UMC Utrecht, Utrecht, The Netherlands; 4 Department of Clinical Epidemiology, Leiden University Medical Center, Leiden, The Netherlands

**Keywords:** Rib fixation, Flail chest, Multiple rib fractures, Long term follow-up

## Abstract

**Purpose:**

Rib fixation for flail chest has been shown to improve in-hospital outcome, but little is known about treatment for multiple rib fractures and long-term outcome is scarce. The aim of this study was to describe the safety, long-term quality of life, and implant-related irritation after rib fixation for flail chest and multiple rib fractures.

**Methods:**

All adult patients with blunt thoracic trauma who underwent rib fixation for flail chest or multiple rib fractures between January 2010 and December 2016 in our level 1 trauma facility were retrospectively included. In-hospital characteristics and implant removal were obtained via medical records and long-term quality of life was assessed over the telephone.

**Results:**

Of the 864 patients admitted with ≥ 3 rib fractures, 166 (19%) underwent rib fixation; 66 flail chest patients and 99 multiple rib fracture patients with an ISS of 24 (IQR 18–34) and 21 (IQR 16–29), respectively. Overall, the most common complication was pneumonia (*n* = 58, 35%). Six (9%) patients with a flail chest and three (3%) with multiple rib fractures died, only one because of injuries related to the thorax. On average at 3.9 years, follow-up was obtained from 103 patients (62%); 40 with flail chest and 63 with multiple rib fractures reported an EQ-5D index of 0.85 (IQR 0.62–1) and 0.79 (0.62–0.91), respectively. Forty-eight (48%) patients had implant-related irritation and nine (9%) had implant removal.

**Conclusions:**

We show that rib fixation is a safe procedure and that patients reported a relative good quality of life. Patients should be counseled that after rib fixation approximately half of the patients will experience implant-related irritation and about one in ten patients requires implant material removal.

**Electronic supplementary material:**

The online version of this article (10.1007/s00068-018-1009-5) contains supplementary material, which is available to authorized users.

## Background

Chest trauma is currently the second leading cause of trauma-related death and multiple rib fractures are the most common injury in these patients [1]. Due to the impact of pulmonary complications, flail chest and multiple rib fractures are still associated with a 10–22% mortality rate with increasing rates for every additional rib involved [2].

Conservative treatment for rib fractures is considered the gold standard and consists of mechanical ventilation (if indicated), pulmonary hygiene, and adequate pain management. In the last century, many different surgical techniques concerning rib fixation were described in the literature without becoming common clinical practice. However, due to technical improvements there is a growing popularity of surgical rib fixation which aims to increase the stability of the chest, lessen chest wall deformity, and improve pulmonary function [3].

In a recent meta-analysis, the authors recommend rib fixation over conservative treatment for adult patients with flail chest to decrease mortality, shorten days on mechanical ventilation, hospital and intensive care length of stay, and decrease incidence of pneumonia and need for tracheostomy [3]. Although rib fixation of patients with flail chest showed promising results, little is known about rib fixation for patients with multiple rib fractures without a flail chest. Furthermore, only few small studies have described the long-term outcome and quality of life after rib fixation [4–7]. Therefore, the aim of this study was to describe the safety, long-term quality of life, and implant-related irritation after rib fixation for flail chest or multiple rib fractures.

## Methods

### Study design and participants

All medical records of patients admitted with rib fractures following blunt thoracic trauma between January 2010 and December 2016 in the University Medical Center Utrecht, a level 1 trauma facility, were retrospectively reviewed. Eligible patients were identified using procedural codes and the Dutch National Trauma Registry. For this study, we included all adult patients with blunt thoracic trauma who underwent rib fixation for flail chest (defined as three or more consecutive ribs fractured in at least two places and clinical signs of paradoxical chest wall movement) or multiple rib fractures (defined as three or more unilateral rib fractures). We did not further distinguish between multiple rib fractures with or without chest deformity due to the retrospective nature of this study. Exclusion criteria were age below 18 years, fewer than three fractured ribs, no availability of an admission CT scan of the chest, and transfer from or to another hospital. Our institutional review board approved a waiver of consent under protocol number 17-914/C.

### Indication for surgery

**Fig. 1:**
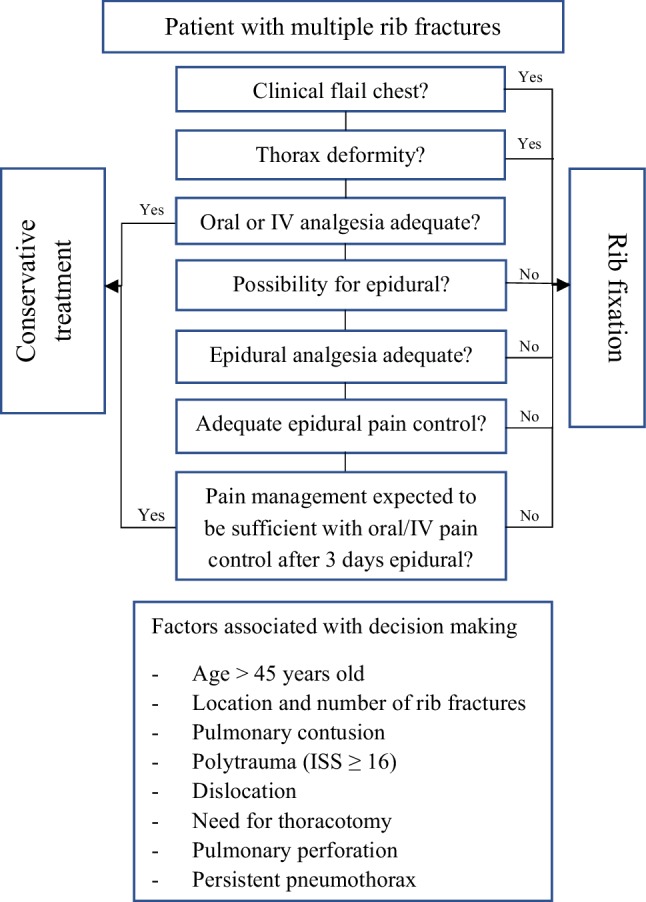
Clinical treatment algorithm for patients with rib fractures

The indication for surgical rib fixation followed from a clinical-based algorithm considering several injuries and patient-specific characteristics as shown in Fig. 1. There was a strict indication for patients with a clinical flail chest (paradoxical breathing). Failure of pain management with tachypnea and dyspnea was considered an indication for surgical rib fixation in patients with multiple rib fractures.

### Patient characteristics at hospital admission

The following characteristics were obtained from medical records based on the recording at admission: age, sex, American Society of Anaesthesiologists (ASA) score, trauma mechanism, Abbreviated Injury Scale (AIS), ISS, Thoracic Trauma Severity Score (TTSS), number of rib fractures, bilateral rib fractures, involvement of the first rib as these are associated with higher impact trauma, rib fractures in the upper/middle/lower third or dorsal side of the thorax, displacement, concomitant injuries as described on the admission CT scan, and blood pH and base excess. The TTSS (range 0–25) is a scoring system that helps to predict thorax-related complications after thoracic trauma and is based on the number of rib fractures, pulmonary contusion, PaO2/FIO2 ratio, pleural involvement, and age [8]. Displacement was defined as a shaft width displacement of the fracture parts in the transversal plane on CT. Dorsal fractures were defined as rib fractures behind the dorsal axillary line.

### Surgical procedure and characteristics

All procedures were performed or supervised by senior trauma surgeons experienced with surgical treatment of rib fractures. Preoperative planning of the procedure was done using chest computed tomography (CT) with 3D reconstructions. Preoperative antibiotic prophylaxis (2 g of Cefazolin) was administered intravenously in all patients. Depending on the site of the fractures, patients were positioned in the supine, lateral or prone position. The surgical approach was performed as described by Taylor [9]. After reduction, internal fixation using the MatrixRIB™ system (Depuy Synthes^®^, Amersfoort, The Netherlands) was performed. Fixation was preferably done with three bicortical screws on each side of the fracture. If plate fixation was not possible due to anatomical boundaries and rib fixation was deemed necessary, splints were used. The number of fixed ribs was at the discretion of the surgeon, and depended on anatomical boundaries and the possibility to regain stability of the chest wall during respiration. Tube thoracostomy was performed in case of pneumothorax or hemothorax at initial presentation or clinical suspicion of pneumothorax during surgery. Postoperative chest radiography was performed in all patients to document surgical result and to rule out early complications. Patients were encouraged to mobilize as soon as possible with the help of physiotherapy and aggressive pain management. All patients had an outpatient department visit 6 weeks after discharge and were counseled to visit if they experienced any thorax-related problems such as pain, dyspnea or irritation.

The following surgery-related characteristics regarding rib fixation were extracted from the medical record: time until surgery, duration of surgery, surgical approach, number of ribs fixated, the ratio of fixated ribs to fractured ribs, side of rib fixation, and fixation of dorsal rib fractures.

### Short- and long-term outcome measures

Short-term outcome measures were hospital length of stay (HLOS), ICU-LOS, duration of invasive mechanical ventilation (IMV), need for tracheostomy, and incidence of surgical complications after rib fixation [e.g., pneumonia, implant-related infection, wound infection, and acute respiratory distress syndrome (ARDS)]. Pneumonia was defined as having clinical signs (fever, coughing, desaturation) requiring antibiotic treatment, with or without positive cultures. Implant-related infection was defined as clinical symptoms (e.g., redness, drainage from surgical wound, fever, pain, elevated CRP, or leukocytes) requiring incision and drainage and intravenous antibiotics following a previously published protocol [10]. ARDS was defined by severe hypoxemia with a PaO2/FIO2 smaller than 100 mm Hg.

Long-term outcome measures were quality of life, number of implant removals due to complications of patient complaints, and level of dyspnea. To assess the long-term outcome measures after rib fixation, patients were contacted by phone after a minimum of 12 months of follow-up. The patient’s contact person and general practitioner were approached for additional contact details if patients could not be reached after a minimum of five phone call attempts.

Quality of life was assessed with the EQ-5D-5L, which is a standardized instrument for generic health status measurement [11]. The EQ-5D-index ranges from − 0.33 to 1.00 where higher scores indicate better quality of life. The EQ-VAS is a patient’s subjective measurement of generic health ranging from 0 to 100, where higher scores represent better subjective health experience. The level of dyspnea was measured with the modified Medical Research Council Dyspnea Scale (mMRC) which is a five-category scale that characterizes the level of dyspnea with physical activity where higher scores corresponds to more dyspnea [12]. Patients who had implant removal were asked for the reason of removal following the algorithm and definitions as described by Hulsmans et al. [13]. Implant removal due to irritation was considered a minimum of 6 months after rib fixation and after discussing the possible harms and benefits with the patient. Apart from the well-known pitfalls after implant removal in general, the most important pitfall of rib implant removal is the risk of a pneumothorax. Therefore, standard chest tube placement should be considered after this procedure. Implant-related irritation at the time of the interview was defined as physical complaints which could be attributed to the implant.

### Statistical analysis

All analyses were performed separately for the groups of patients with flail chest and the group of patients with multiple rib fractures. Baseline characteristics were presented as median and interquartile range (IQR) for continuous variables, and absolute numbers with percentage for categorical variables. The non-parametric outcome measures were normalized with a cubic transformation for left skewed data and a log transformation for HLOS and ICU-LOS. In bivariate analysis, the association of the HLOS, ICU-LOS, and EQ-5D-index with the baseline characteristics was assessed using linear regression. Variables with a *p* value of below 0.05 in this analysis were entered into a multivariable linear regression model to assess their ability to explain the variation in HLOS, ICU-LOS, and quality of life. Given the small dataset with the high number of potential variables a robustness check of the primary multivariable regression model was performed by means of the least shrinkage and selection operator (LASSO) technique [14]. LASSO performs automatic variable selection by shrinking coefficients and giving a penalty for the number of variables in the model. LASSO is considered a robust and objective alternative for the more regularly performed stepwise variable selection for multivariable regression. The two statistical models were compared in terms of the variables that showed a relation with the outcome of interest. All analyses were performed with Stata 13 (StataCorp LP, College Station, TX, USA); a *p* value of less than 0.05 was considered significant.

## Results

**Fig. 2:**
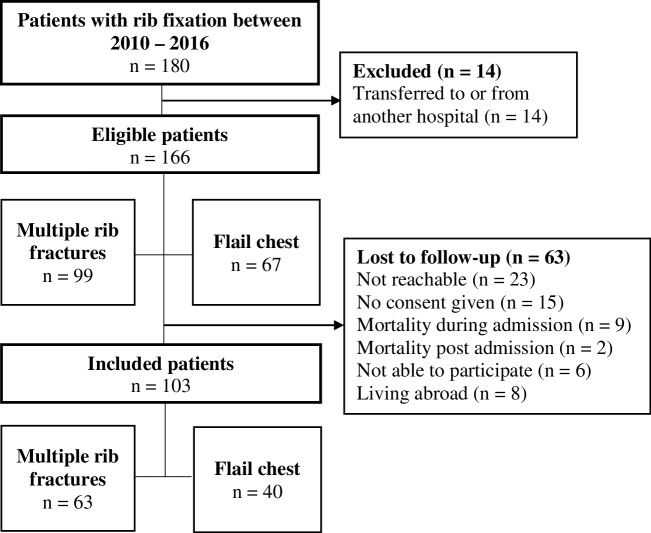
Flowchart of patient inclusion

Between 2010 and 2016, in our hospital, a total of 864 patients were admitted with chest trauma resulting in three or more rib fractures. Ultimately, 166 patients (19%) who underwent rib fixation were included for analysis: 67 with flail chest and 99 with multiple rib fractures (Fig. 2). Of these, 137 (83%) were treated with plate osteosynthesis, 29 (17%) with a combination of plate osteosynthesis and intramedullary splints, and 1 only with intramedullary splints. Outcome information, at a minimum of 12 months after rib fixation, was obtained from 103 patients (62%): 40 with flail chest and 63 with multiple rib fractures.

### Flail chest

The median age of patients with flail chest was 57 (IQR 48–69) years and the majority were male (*n* = 52, 78%) (Table 1). The median ISS was 24 (IQR 18–34) and the median number of fractured ribs was 10 (IQR 8–12). Rib fixation was performed after a median of two (IQR 1–3) days and the ratio of fixated ribs to fractured ribs was 0.49 (Table 2).

**Table 1:** Baseline characteristics of patient with rib fixation for flail chest or multiple rib fractures

Variable	Flail chest	Multiple rib fractures
	*n* = 67	*n* = 99
Age (median, IQR)	57 (48–69)	56 (47–64)
Male (*n*, %)	52 (78)	81 (82)
ASA score (*n*, %)		
1–2	57 (92)	82 (84)
> 2	5 (8)	16 (16)
Trauma mechanism (*n*, %)		
Motor vehicle accident	25 (37)	33 (33)
Fall from height/stairs	17 (25)	29 (29)
Other	25 (37)	37 (37)
AIS (median, IQR)		
Head	0 (0–3)	0 (0–2)
Face	0 (0–0)	0 (0–0)
Thorax	4 (3–4)	4 (3–4)
Abdomen	0 (0–2)	0 (0–2)
Extremities	2 (0–3)	2 (0–2)
ISS (median, IQR)	24 (18–34)	21 (16–29)
TTSS (median, IQR)	13 (11–15)	10 (8–12)
No. of rib fractures (median, IQR)	10 (8–12)	7 (6–10)
Bilateral rib fractures (*n*, %)	26 (39)	34 (34)
First rib fracture (*n*, %)		
Unilateral	18 (27)	16 (16)
Bilateral	7 (10)	11 (11)
Location rib fracture (*n*, %)		
Costae 1–4	62 (93)	84 (85)
Costae 5–8	67 (100)	99 (100)
Costae 9–12	46 (69)	60 (61)
Displacement (*n*, %)	47 (70)	58 (59)
Dorsal fracture (*n*, %)	59 (88)	67 (68)
Concomitant injuries (*n*,%)		
Pulmonary contusion	44 (66)	43 (43)
Pneumothorax	50 (75)	66 (67)
Hemothorax	16 (24)	21 (21)
Sternum fracture	7 (10)	16 (16.2)
Blood pH (median, IQR)	7.3 (7.28–7.4)	7.4 (7.3–7.4)
Base excess (median, IQR)	− 2 (− 5 to − 1)	− 1 (− 3.5 to 0.7)

*ASA* American Society of Anesthesiologists, *ISS* Injury Severity Score, *TTSS* Thoracic Trauma Severity Score, *AIS* Abbreviated Injury Score, *IQR* interquartile range

**Table 2:** Surgery-related characteristics

Variable	Flail chest	Multiple rib fractures
	*n* = 67	*n* = 99
Time until surgery (days, median, IQR)	2 (1–3)	2 (1–4)
Duration of surgery (minutes, median, IQR)	130 (91–155)	98 (71–122)
Surgical approach (*n*, %)		
Anterior	9 (13)	12 (12)
Anterolateral	9 (13)	17 (17)
Posterior	10 (15)	19 (19)
Posterolateral	32 (48)	39 (39)
Combination	7 (10)	12 (12)
No. of ribs fixated (median, IQR)	4 (4–6)	4 (3–5)
No. of ribs fixated/total ribs fractured (median, IQR)	0.5 (0.36–0.6)	0.5 (0.38–0.67)
Side of rib fixation (*n*, %)		
Left	34 (51)	45 (46)
Right	26 (39)	46 (47)
Bilateral	7 (10)	8 (8)
Fixation of dorsal fractures (*n*, %)	35 (52)	36 (36)

*IQR* interquartile range, *ICU* intensive care unit, *IMV* invasive mechanical ventilation

Among patients with flail chest, the most common complication was pneumonia (*n* = 26, 39%) followed by excess pleural fluid (*n* = 3, 5%) and implant-related infection (*n* = 2, 3%) (Table 3). One patient had a tension pneumothorax perioperatively and required a chest tube. Six (9%) patients died during hospital admission; all were because of concomitant injuries that were not related to the rib fractures. Two patients had an infaust neurological prognosis, one patient died of cardiac failure, one patient developed secondary bacterial meningitis, and one patient with metastasized carcinoma and IC acquired weakness wished no further treatment.

**Table 3:** In-hospital complications after rib fixation

In-hospital complications	Flail chest (*n*,%)	Multiple rib fractures (*n*,%)
	*n* = 47	*n* = 53
Pneumonia	26 (39)	32 (32)
Excess pleural fluid	3 (4.5)	3 (3)
Implant-related infection	2 (3)	3 (3)
Hemothorax	2 (3)	2 (2)
Pneumothorax	2 (3)	2 (2)
Tension pneumothorax	1 (1)	2 (2)
ARDS	2 (3)	3 (3)
Postoperative bleeding	1 (1.5)	1 (1)
Wound infection	1 (1.5)	0 (0)
Pleural empyema	1 (1.5)	0 (0)
Hematoma	0 (0)	1 (1)
Revision of dislocated splints	0 (0)	1 (1)
In-hospital mortality	6 (9)	3 (3)

*ARDS* acute respiratory distress syndrome

**Fig. 3:**
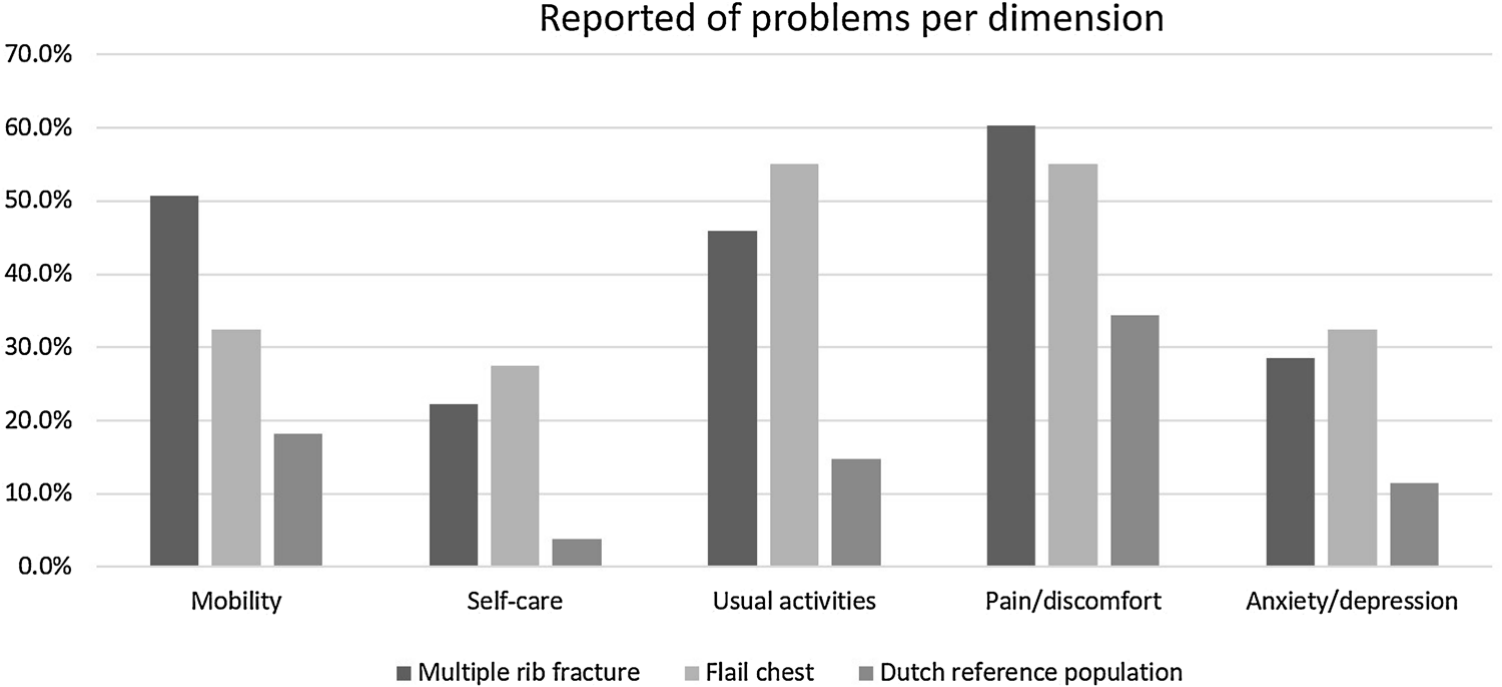
EQ-5D-5L-reported problems per domain

The median HLOS was 19 (11–26) days and 44 (66%) patients required ICU admission with a median ICU-LOS 8 (6–14) days (Table 4). The median follow-up duration was 3.1 years (IQR 2.4–5.1; range 1–7.5) and 40 (60%) patients were available for follow-up. The median quality of life as measured with the EQ-5D index at follow-up was 0.85 (IQR 0.62–1) with an EQ-VAS of 75 (IQR 63–85). Figure 3 shows the proportion of patients reporting problems specified per EQ-5D domain. Twenty-one (53%) patients reported implant-related irritation. Five (13%) patients had their implant removed due to irritation on average 1.1 (range 0.64–1.6) years after rib fixation. Patients reporting implant-related irritation at the time of the interview had a significant lower median EQ-5D index compared to patients without implant-related irritation (*z* = 2.97; *p* = 0.003). Eleven patients (28%) reported mild to severe complaints of dyspnea.

**Table 4:** Outcome measures after rib fixation for flail chest or multiple rib fractures

Short-term outcome measures	Flail chest	Multiple rib fractures
	*n* = 67	*n* = 99
HLOS (days, median, IQR)	19 (11–26)	14 (10–28)
ICU admission (*n*, %)	44 (66)	44 (44)
ICU-LOS among those admitted to ICU (days, median, IQR)	8 (6–14)	9 (2–16)
Number of patient with IMV (*n*, %)	40 (60)	35 (35)
Duration of IMV among those ventilated (days, median, IQR)	6 (4–12)	9 (4–16)
Tracheostomy (*n*, %)	7 (10)	9 (9)

Long-term outcome measures	*n* = 40	*n* = 63
EQ-5D index (median, IQR)	0.85 (0.62–1)	0.79 (0.62–0.91)
EQ VAS (median, IQR)	75 (63–85)	73 (65–80)
Implant-related irritation (*n*, %)	21 (53)	28 (44)
Implant removed (*n*, %)	5 (13)	4 (6)
Reason removed (*n*, %)		
Attributable to implant-related irritation	5 (13)	4 (6)
Patient’s wish or surgeon’s preference	0 (0)	0 (0)
Status not removed (*n*, %)		
No irritation	19 (47)	35 (56)
Experiencing irritation, but implant removal not necessary	12 (30)	11 (18)
Experiencing irritation, but no request for removal owing to fear of reoperation	1 (3)	2 (3)
Experiencing irritation, considering removal	3 (8)	10 (16)
Revision implant (*n*, %)	1 (3)	1 (2)
mMRC (*n*, %)		
0	17 (43)	31 (49)
1	12 (30)	23 (37)
2	6 (15)	5 (8)
3	4 (10)	3 (5)
4	1 (3)	1 (2)
Follow-up duration in years (median, IQR)	3.1 (2.4–5.1)	4.4 (3.4–5.9)
Follow-up range duration in years (min, max)	1–7.5	1–7.6

*HLOS* hospital length of stay, *ICU-LOS* intensive care unit length of stay, *IQR* interquartile range, *IMV* invasive mechanical ventilation, *mMRC* modified Medical Research Council

The association between patient characteristics and the outcomes are presented in Appendix 1. In multivariable linear regression, male sex and sternum fracture appeared to be independently associated with the EQ-5D index (Appendix 2). We did not observe an association with HLOS. A higher AIS-head appeared to be associated with ICU-LOS. The associations found in the three multivariable models were also found when applying LASSO, indicating robustness of the models.

### Multiple rib fractures

The median age of the 99 patients with multiple rib fractures was 56 (IQR 47–64) years and the majority were male (*n* = 82, 82%) (Table 1). The median ISS was 21 (IQR 16–29) and the median number of fractured ribs was 7 (IQR 6–10). Surgery was performed after a median of two (IQR 1–4) days and the ratio of fixated ribs to fractured ribs was 0.52 (Table 2).

Among patients operated on multiple rib fractures, pneumonia was the most common complication (*n* = 32, 32%) followed by excess pleural fluid (*n* = 3, 3%) and implant-related infection (*n* = 3, 3%) (Table 3). Two (2%) patients suffered a tension pneumothorax postoperatively and were successfully treated with a chest tube. One (1%) patient needed revision surgery due to two dislocated intramedullary splints resulting in a hemothorax. Three (3%) patients died during hospital admission: one because of respiratory failure possibly associated with the suffered rib fractures and the other two as a result of concomitant injuries not related to the thorax. One had unmanageable infectious episodes from unknown origin and did not want further treatment. One patient had a systemic inflammatory response with decompensated liver cirrhosis, kidney failure, and developed acute respiratory distress syndrome (ARDS).

The median HLOS was 14 (IQR 10–28) days and 44 patients (44%) required ICU admission with a median ICU-LOS of 9 (IQR 2–16) days (Table 4). The median follow-up was 4.4 years (IQR 3.4–5.9; range 1–7.6) and 63 patients (63%) were available for follow-up. The median quality of life as measured with the EQ-5D index at follow-up was 0.79 (IQR 0.62–0.91) with an EQ-VAS of 73 (IQR 65–80). Figure 3 shows the proportion of patients reporting problems specified per EQ-5D domain. After rib fixation for multiple rib fractures, 28 (44%) of the patients experienced implant-related irritation. Four patients (6.3%) had their implant removed due to irritation on average 1.8 (range 0.91–4.2) years after rib fixation. Patients reporting implant-related irritation at the time of the interview had a significant lower median EQ-5D index compared to patients without implant-related irritation (*z* = 3.30; *p* = 0.001). Nine patients (14%) reported mild to serious complaints of dyspnea.

The association between each patient characteristic and the outcomes are presented in Appendix 3. In multivariable regression, we did not observe an association of the EQ-5D index and the baseline characteristics (Appendix 4). A higher AIS-head, AIS-extremities, and AIS-abdomen appeared to be associated with HLOS. A higher AIS-face, AIS-extremities, and base excess appeared to be associated with ICU-LOS. The associations found in the three multivariable models were also found when applying LASSO.

## Discussion

In this cohort study of 166 patients admitted to a Dutch level 1 trauma facility, the reported quality of life was relatively good after rib fixation for flail chest or multiple rib fractures at a median follow-up of 3.1 and 4.4 years, respectively. A mortality rate of 5% was demonstrated in this cohort. Approximately half of the patients experienced implant-related irritation after rib fixation and about 10 percent had the implant material, or part of it, removed due to this irritation. At follow-up, 15–18% of the patients reported mild tot serious complaints of dyspnea as measured with the mMRC.

In our cohort, the mortality rate for patients with flail chest was 9% and for multiple rib fractures 3%; only one death could be directly ascribed as the consequence of the suffered rib fractures. There were three important surgery-related complications resulting in a tension pneumothorax; all were successfully treated with a chest tube. The low mortality rate as well as the low number of surgical complications indicate the relative safety of this procedure in this patient cohort. The most frequent complication was pneumonia in 39% of the patients with flail chest and 32% of the patients with multiple rib fractures and is comparable with the existing literature. However, definitions used for pneumonia differ in the literature making this outcome measure difficult to compare across studies. The incidence of ARDS was 3% in both groups and was low compared to an ARDS incidence of 13% in a previously published cohort of polytrauma patients, predominantly chest trauma, from our hospital [15]. This low rate of ARDS in our cohort could be attributed to the effects of rib fixation. The rate of implant-related infection was 3% in our cohort and was similar to the infection rate reported by Pieracci et al. [16] in a similar but smaller cohort.

The duration of mechanical ventilation and ICU-LOS among patients admitted to the ICU in our cohort were comparable or shorter than the three RCTs available on this subject [17–19]. Another interesting finding in our study was that injury severity, as defined by the Abbreviated Injury Scale, in other body regions such as head, face, abdomen, and extremities was associated with a longer HLOS and/or ICU-LOS, while no association was seen with injury severity of the thorax. One explanation could be that rib fixation successfully minimized the impact of chest injury on the outcome measures. ICU-LOS and HLOS are frequently used to measure the success of rib fixation and it should be kept in mind that a small but potential beneficial effect could be masked by associated injury when comparing different treatment strategies for rib fractures. This emphasizes the necessity of sufficient group sizes when comparing treatment strategies in this often-heterogeneous group of patients; nonetheless, there is a lack of large patient series in the current literature.

The quality of life in our study, a EQ-5D index of 0.85 for patients with flail chest and 0.79 for patients with multiple rib fractures, is comparable to the Dutch reference population index of 0.87 [20] and compared to studies describing different polytrauma cohorts these results were good [21–24]. There was no difference in quality of life between patients with flail chest and patients with multiple rib fractures as both indices were within the range of the minimal clinically important difference for the EQ-5D (the minimal score difference detectable by the patient) [25, 26]. Although, one might expect a worse outcome for flail chest patients compared to patients with multiple rib fractures, in this cohort, patients with multiple rib fractures had similar injury severity scores which might explain comparability. Caragounis et al. presented comparable results after 1-year follow-up of 45 patients with rib fixation for flail chest and multiple rib fractures with an EQ-5D index of 0.93 [27]. Similar results were reported by Mayberry et al. [4] in a cohort of 15 patients after rib fixation. In another study, Campbell et al. [6] reported on quality of life of 20 patients more than 1 year after rib fixation and showed a lower quality of life as compared to the reference population possibly due to the higher ISS scores in this patient cohort. There were a high number of reported problems per domain ranging from 22 to 60%, with the most substantial limitation experienced in the domain of pain and discomfort. It cannot be extracted from the EQ-5D-5L if the pain is situated in the chest area. Farquhar et al. [28] reported the EQ-5D-5L of 11 patients with rib fixation for flail chest at an unspecified long-term follow-up, and reported a slightly higher number of problems per domain as compared to our results, but also found the highest rate of problems in the domain of pain and discomfort. Although residual pain and chest stiffness are commonly reported in the literature, patient satisfaction is high after rib fixation at long-term follow-up [5–7].

Implant removal after rib fixation is a challenging and time-consuming procedure. Due to the angular stable system and soft titanium, we encountered several technical problems during implant removal. In one case, a grinding machine was used to remove plate and screwheads leaving the body of the screws in place. In other cases, a diamond drill was used to remove the screwhead from the plate also leaving the screw body behind. Because implant removal is challenging, perforation of the pleura happens easily. Therefore, a chest tube should be considered after implant removal.

Two of the three clinical trials in this field performed rib fixation on patients with flail chest who were ventilator dependent without prospect of successful weaning. All three studies had different strict exclusion criteria such as severe injuries to other body systems, head trauma, or patients who did not develop acute respiratory failure [17–19]. Because of the heterogenicity in the aforementioned clinical trials, no clear indication for rib fixation has been defined. Also, very few studies have enrolled any substantial number of patients with multiple rib fractures without flail chest making the indication for these patients unknown. We made use of a clinical treatment algorithm (Fig. 1) based on the previous literature and experience in our hospital, which provides guidance in decision-making for both patients with flail chest and patients with multiple rib fractures.

In addition to the right indication, timing of the procedure is of major importance. The main reason for rib fixation is to stabilize the thorax to increase pulmonary mechanics and reduce pain. In a recently published study, Pieracci et al. [29] concluded that early surgical stabilization was indeed associated with favorable outcome. Additionally, they found that late surgical stabilization resulted in a significantly longer operating time for the same type of rib fracture. They hypothesized that this could be ascribed to tissue inflammation resulting in obscured planes and increased bleeding. Therefore, in our hospital, rib fixation is performed according to the treatment algorithm but preferably as early as possible after hospital admission.

The results should be interpreted in the light of several limitations. First, the EQ-5D-5L and mMRC are subjective questionnaires and assess general health and not specifically thorax-related problems. The vast majority of the patients described in this cohort were polytrauma patients; therefore, concomitant injuries but also comorbidities could have influenced the outcome. Second, due to the retrospective nature, this study could be subject to data loss and underreporting of complications. Consequently, no data were available on quality of life of patients before implant removal to objectify any improvement, although no differences were observed after implant removal compared with the rest of the patients. Third, follow-up differed per patient and ranged from 1 to 7.5 years. We assumed that for the majority of patients quality of life will improve most significantly in the first year after trauma and to a lesser extent thereafter, which is supported by our finding that there was no association between follow-up duration and quality of life (Spearman’s rho 0.14; *p* = 0.164). Fourth, rib fixation was performed following the incision of a thoracotomy in the earlier years which gradually changed to a more minimally invasive approach in the following years. Nonetheless, there was no correlation between year of surgery and the outcome measures. Finally, the Dutch reference values for the EQ-5D were obtained from the three-category EQ-5D version whereas our results were measured using the newer five-category version. The additional answer categories provide the possibility for the patient to report milder problems which could have resulted in a higher percentage of reported problems as compared to the available Dutch reference population.

This is the largest study to present the long-term follow-up of patients after rib fixation following a clear clinical treatment algorithm. We show that rib fixation is a safe treatment option for both patients with flail chest and patients with multiple rib fractures and that patients report a relatively good quality of life at long-term follow-up as compared to the Dutch reference population. Patients should be counseled that after rib fixation approximately half of the patients will experience implant-related irritation and about one in ten patients requires implant material removal due to this irritation. Future studies should focus on further development of the indication for rib fixation and should aim to identify the patient who will benefit most from rib fixation.

## Electronic supplementary material

Below is the link to the electronic supplementary material.

Supplementary material 1 (DOCX 30 KB)
