# Supplementary material for: Long-term follow-up after rib fixation for flail chest and multiple rib fractures
Source: Eur J Trauma Emerg Surg. 2018 Sep 18;45(4):645–54. doi: 10.1007/s00068-018-1009-5 (PMC6689022; doi:10.1007/s00068-018-1009-5)
Supplement: Supplementary file 1 — Supplementary material 1 (DOCX 30 KB) [file 68_2018_1009_MOESM1_ESM.docx]

| **Appendix 1.** Bivariate analysis of the baseline characteristics and the outcome measures of patients with flail chest | | | | | | | | | |
| --- | --- | --- | --- | --- | --- | --- | --- | --- | --- |
| Variable | EQ-5D index | | | HLOS | | | ICU length of stay | | |
|  | coefficient | se | p | coefficient | se | p | coefficient | se | p |
| Age | 0.000 | 0.001 | 0.837 | 0.002 | 0.006 | 0.705 | 0.018 | 0.010 | 0.085 |
| Male | 0.080 | 0.033 | ***0.019*** | -0.130 | 0.206 | 0.530 | 0.200 | 0.366 | 0.586 |
| ASA-score | 0.044 | 0.090 | 0.625 | 0.716 | 0.312 | ***0.025*** | 1.400 | 0.557 | ***0.015*** |
| Trauma mechanism | -0.017 | 0.017 | 0.332 | -0.141 | 0.098 | 0.156 | -0.102 | 0.177 | 0.566 |
| AIS |  |  |  |  |  |  |  |  |  |
| Head | -0.007 | 0.012 | 0.542 | 0.088 | 0.056 | 0.121 | 0.275 | 0.096 | ***0.005*** |
| Face | 0.020 | 0.017 | 0.257 | 0.080 | 0.109 | 0.465 | 0.055 | 0.195 | 0.780 |
| Thorax | -0.010 | 0.021 | 0.640 | 0.070 | 0.115 | 0.544 | 0.319 | 0.200 | 0.116 |
| Abdomen | 0.000 | 0.011 | 0.987 | 0.084 | 0.065 | 0.200 | 0.209 | 0.114 | 0.072 |
| Extremities | -0.013 | 0.011 | 0.227 | 0.187 | 0.061 | ***0.003*** | 0.493 | 0.099 | ***0.000*** |
| ISS | -0.002 | 0.001 | 0.232 | 0.019 | 0.007 | ***0.013*** | 0.057 | 0.012 | ***0.000*** |
| TTSS | -0.005 | 0.007 | 0.504 | 0.022 | 0.033 | 0.498 | 0.193 | 0.051 | ***0.000*** |
| No. of rib fractures | -0.002 | 0.004 | 0.610 | 0.059 | 0.021 | ***0.007*** | 0.089 | 0.038 | ***0.024*** |
| Bilateral rib fractures | -0.047 | 0.030 | 0.121 | 0.509 | 0.165 | ***0.003*** | 0.678 | 0.302 | ***0.028*** |
| First rib fracture | 0.005 | 0.021 | 0.807 | 0.260 | 0.126 | ***0.043*** | 0.232 | 0.227 | 0.312 |
| Location rib fracture |  |  |  |  |  |  |  |  |  |
| Costae 1 - 4 | -0.023 | 0.058 | 0.693 | 0.669 | 0.323 | ***0.042*** | -0.136 | 0.585 | 0.817 |
| Costae 5 - 8 | NA | NA | NA | NA | NA | NA | NA | NA | NA |
| Costae 9 - 12 | 0.011 | 0.031 | 0.718 | -0.025 | 0.190 | 0.897 | 0.501 | 0.328 | 0.132 |
| Displacement | -0.056 | 0.030 | 0.071 | 0.091 | 0.192 | 0.636 | 0.173 | 0.337 | 0.610 |
| Dorsal fracture | 0.005 | 0.046 | 0.920 | -0.287 | 0.268 | 0.288 | -0.374 | 0.473 | 0.431 |
| Concomitant injuries |  |  |  |  |  |  |  |  |  |
| Lung contusion | -0.009 | 0.032 | 0.769 | 0.329 | 0.177 | 0.067 | 0.487 | 0.317 | 0.129 |
| Pneumothorax | -0.030 | 0.036 | 0.414 | 0.068 | 0.198 | 0.732 | -0.010 | 0.352 | 0.977 |
| Hemothorax | 0.018 | 0.034 | 0.593 | -0.149 | 0.201 | 0.461 | -0.228 | 0.358 | 0.526 |
| Sternum fracture | -0.103 | 0.048 | ***0.038*** | 0.472 | 0.276 | 0.091 | 1.255 | 0.475 | ***0.010*** |
| Blood pH | 0.025 | 0.142 | 0.863 | -2.167 | 0.836 | ***0.012*** | -4.863 | 1.412 | ***0.001*** |
| Base Excess | 0.001 | 0.004 | 0.904 | -0.083 | 0.024 | ***0.001*** | -0.174 | 0.041 | ***0.000*** |

| **Appendix 2.** Bivariate analysis of the baseline characteristics and the outcome measures of patients with multiple rib fractures | | | | | | | | | |
| --- | --- | --- | --- | --- | --- | --- | --- | --- | --- |
| Variable | EQ-5D index | | | HLOS | | | ICU length of stay | | |
|  | coefficient | se | p | coefficient | se | p | coefficient | se | p |
| Age | 0.000 | 0.001 | 0.767 | 0.004 | 0.005 | 0.414 | 0.005 | 0.009 | 0.600 |
| Male | -0.016 | 0.044 | 0.713 | -0.301 | 0.181 | 0.100 | -0.288 | 0.322 | 0.373 |
| ASA-score | -0.016 | 0.049 | 0.736 | 0.078 | 0.191 | 0.683 | 0.127 | 0.334 | 0.705 |
| Trauma mechanism | 0.012 | 0.018 | 0.513 | -0.094 | 0.084 | 0.265 | -0.070 | 0.148 | 0.639 |
| AIS |  |  |  |  |  |  |  |  |  |
| Head | 0.008 | 0.013 | 0.545 | 0.220 | 0.043 | ***0.000*** | 0.340 | 0.077 | ***0.000*** |
| Face | 0.015 | 0.024 | 0.516 | 0.232 | 0.090 | ***0.012*** | 0.522 | 0.155 | ***0.001*** |
| Thorax | 0.020 | 0.021 | 0.362 | 0.091 | 0.104 | 0.382 | 0.014 | 0.183 | 0.941 |
| Abdomen | -0.008 | 0.011 | 0.492 | 0.187 | 0.048 | ***0.000*** | 0.315 | 0.085 | ***0.000*** |
| Extremities | 0.007 | 0.013 | 0.602 | 0.259 | 0.049 | ***0.000*** | 0.368 | 0.091 | ***0.000*** |
| ISS | 0.001 | 0.002 | 0.488 | 0.037 | 0.006 | ***0.000*** | 0.056 | 0.010 | ***0.000*** |
| TTSS | 0.006 | 0.006 | 0.308 | 0.059 | 0.025 | ***0.019*** | 0.122 | 0.042 | ***0.005*** |
| No. of rib fractures | -0.001 | 0.005 | 0.847 | 0.047 | 0.023 | ***0.042*** | 0.088 | 0.040 | ***0.028*** |
| Bilateral rib fractures | -0.025 | 0.031 | 0.424 | 0.376 | 0.144 | ***0.011*** | 0.627 | 0.254 | ***0.015*** |
| First rib fracture | 0.005 | 0.021 | 0.809 | 0.111 | 0.106 | 0.297 | 0.252 | 0.185 | 0.177 |
| Location rib fracture |  |  |  |  |  |  |  |  |  |
| Costae 1 - 4 | -0.038 | 0.042 | 0.370 | 0.065 | 0.205 | 0.752 | -0.273 | 0.359 | 0.449 |
| Costae 5 - 8 | NA | NA | NA | NA | NA | NA | NA | NA | NA |
| Costae 9 - 12 | -0.026 | 0.032 | 0.421 | 0.171 | 0.151 | 0.260 | 0.164 | 0.266 | 0.540 |
| Displacement | -0.007 | 0.031 | 0.812 | 0.119 | 0.147 | 0.421 | 0.397 | 0.256 | 0.124 |
| Dorsal fracture | -0.008 | 0.032 | 0.802 | 0.082 | 0.156 | 0.603 | 0.083 | 0.275 | 0.765 |
| Concomitant injuries |  |  |  |  |  |  |  |  |  |
| Lung contusion | 0.009 | 0.032 | 0.786 | 0.255 | 0.141 | 0.073 | 0.547 | 0.245 | ***0.028*** |
| Pneumothorax | 0.016 | 0.032 | 0.619 | 0.233 | 0.148 | 0.121 | 0.197 | 0.263 | 0.456 |
| Hemothorax | 0.006 | 0.038 | 0.868 | 0.095 | 0.173 | 0.586 | 0.150 | 0.304 | 0.623 |
| Sternum fracture | -0.043 | 0.040 | 0.289 | -0.054 | 0.192 | 0.779 | -0.139 | 0.338 | 0.682 |
| Blood pH | -0.212 | 0.208 | 0.314 | -2.102 | 0.700 | ***0.003*** | -5.739 | 1.138 | ***0.000*** |
| Base Excess | -0.003 | 0.005 | 0.508 | -0.055 | 0.016 | ***0.001*** | -0.139 | 0.026 | ***0.000*** |

| **Appendix 3.** Multivariable linear regression of the baseline characteristics and the outcome measures of patients with flail chest | | | | | |
| --- | --- | --- | --- | --- | --- |
| model for EQ-5D index | coefficient | se | 95% CI | | p |
| Male | 0.080 | 0.031 | 0.017 | 0.142 | ***0.014*** |
| Sternum facture | -0.103 | 0.045 | -0.193 | -0.012 | ***0.027*** |
| model for HLOS |  |  |  |  |  |
| ASA | 0.523 | 0.293 | -0.065 | 1.111 | 0.080 |
| AIS extremities | 0.120 | 0.067 | -0.014 | 0.255 | 0.078 |
| No. of rib fractures | 0.017 | 0.029 | -0.040 | 0.075 | 0.543 |
| Bilateral fractures | 0.192 | 0.239 | -0.287 | 0.672 | 0.425 |
| First rib fracture | 0.146 | 0.146 | -0.146 | 0.439 | 0.320 |
| Base excess | -0.051 | 0.027 | -0.105 | 0.002 | 0.060 |
| model for ICU length of stay |  |  |  |  |  |
| ASA | 0.870 | 0.530 | -0.202 | 1.941 | 0.109 |
| ISS | -0.008 | 0.020 | -0.048 | 0.033 | 0.701 |
| TTSS | 0.088 | 0.060 | -0.033 | 0.209 | 0.150 |
| AIS extremities | 0.226 | 0.136 | -0.050 | 0.502 | 0.105 |
| AIS head | 0.237 | 0.107 | 0.020 | 0.454 | ***0.033*** |
| No. of rib fractures | -0.051 | 0.057 | -0.166 | 0.063 | 0.370 |
| Bilateral fractures | 0.125 | 0.432 | -0.749 | 0.998 | 0.774 |
| Sternum facture | 0.803 | 0.740 | -0.694 | 2.301 | 0.284 |
| Base excess | -0.094 | 0.053 | -0.201 | 0.012 | 0.081 |

| **Appendix 4.** Multivariable linear regression of the baseline characteristics and the outcome measures of patients with multiple rib fractures | | | | | |
| --- | --- | --- | --- | --- | --- |
| model for HLOS | coefficient | se | 95% CI | | p |
| TTSS | 0.032 | 0.021 | -0.011 | 0.074 | 0.143 |
| AIS head | 0.133 | 0.045 | 0.043 | 0.223 | ***0.004*** |
| AIS face | 0.112 | 0.084 | -0.054 | 0.279 | 0.183 |
| AIS extremities | 0.183 | 0.049 | 0.086 | 0.279 | ***0.000*** |
| AIS abdomen | 0.105 | 0.047 | 0.013 | 0.198 | ***0.026*** |
| No. of rib fractures | -0.001 | 0.025 | -0.051 | 0.049 | 0.963 |
| Bilateral fractures | 0.036 | 0.157 | -0.277 | 0.350 | 0.818 |
| Base excess | -0.030 | 0.018 | -0.066 | 0.006 | 0.098 |
| model for ICU-LOS |  |  |  |  |  |
| AIS head | 0.139 | 0.079 | -0.019 | 0.296 | 0.083 |
| AIS face | 0.363 | 0.142 | 0.080 | 0.647 | ***0.013*** |
| AIS extremities | 0.193 | 0.083 | 0.029 | 0.358 | ***0.022*** |
| AIS abdomen | 0.129 | 0.079 | -0.029 | 0.287 | 0.109 |
| TTSS | 0.066 | 0.040 | -0.013 | 0.145 | 0.100 |
| No. of rib fractures | 0.010 | 0.043 | -0.075 | 0.096 | 0.811 |
| Pulmonary contusion | 0.163 | 0.229 | -0.293 | 0.618 | 0.478 |
| Bilateral fractures | -0.132 | 0.267 | -0.664 | 0.401 | 0.623 |
| Base excess | -0.113 | 0.030 | -0.173 | -0.052 | ***0.000*** |
